# Effect of a Brief Heat Exposure on Blood Pressure and Physical Performance of Older Women Living in the Community—A Pilot-Study

**DOI:** 10.3390/ijerph111212623

**Published:** 2014-12-05

**Authors:** Anja Stotz, Kilian Rapp, Juha Oksa, Dawn A. Skelton, Nina Beyer, Jochen Klenk, Clemens Becker, Ulrich Lindemann

**Affiliations:** 1 Department of Clinical Gerontology and Rehabilitation, Robert-Bosch-Hospital, 70376 Stuttgart, Germany; E-Mails: anja.stotz@gmx.de (A.S.); kilian.rapp@rbk.de (K.R.); jochen.klenk@rbk.de (J.K.); clemens.becker@rbk.de (C.B.); 2 Physical Work Capacity Team, Finnish Institute of Occupational Health, 90220 Oulu, Finland; E-Mail: juha.oksa@ttl.fi; 3 Institute of Applied Health Research, Glasgow Caledonian University, Scotland, G4 0BA, UK; E-Mail: dawn.skelton@gcu.ac.uk; 4 Musculoskeletal Rehabilitation Research Unit, Bispebjerg and Frederiksberg Hospitals, University of Copenhagen, 2400 NV Copenhagen, Denmark; E-Mail: ninabeyer.privat@gmail.com; 5 Institute of Epidemiology, Ulm University, 89081 Ulm, Germany

**Keywords:** aerobic capacity, blood pressure, heat, older women

## Abstract

Global climate change is affecting health and mortality, particularly in vulnerable populations. High ambient temperatures decrease blood pressure (BP) in young and middle aged adults and may lead to orthostatic hypotension, increasing the risk of falls in older adults. The aim of this study was to evaluate the feasibility of a test protocol to investigate BP response and aerobic capacity of older adults in a hot indoor environment. BP response and aerobic capacity were assessed in 26 community-dwelling older women (median age 75.5 years) at a room temperature of either 20 °C or 30 °C. The protocol was well tolerated by all participants. In the 30 °C condition systolic and diastolic BP (median difference 10 and 8 mmHg, respectively) and distance walked in 6 min (median difference 29.3 m) were lower than in the 20 °C condition (all *p* < 0.01). Systolic BP decreased after standing up from a lying position in the 30 °C (17.4 mmHg) and 20 °C (14.2 mmHg) condition (both *p* < 0.001). In conclusion, the protocol is feasible in this cohort and should be repeated in older adults with poor physical performance and impaired cardio-vascular response mechanisms. Furthermore, aerobic capacity was reduced after exposure to hot environmental temperatures, which should be considered when recommending exercise to older people during the summer months.

## 1. Introduction

Global climate change, associated with excessive extreme temperatures in winter and summer, is expected to affect human health, particularly in vulnerable populations [1]. As yet there are few recommendations on how to adapt to these changing conditions or how to counteract detrimental effects with regard to health care strategies or environmental solutions [2]. Heat waves have resulted in increased mortality, especially among older adults, in Europe [3,4] and worldwide [5,6,7]. Discussed pathophysiological mechanisms are cardiovascular stress, the inability to increase cardiac output, dehydration and medications, such as diuretics or anticholinergic drugs which aggravate dehydration or suppress sweating [8]. The cardiovascular-related deaths seem to be the most relevant [9]; the impact of other pathophysiological mechanisms is less clear.

High ambient temperatures decrease systolic blood pressure (BP) in young and middle aged people [10]. An additional drop in BP due to orthostatic stress may lead to orthostatic hypotension with an increase in dizziness, fainting and falls in older adults [11,12]. A physiological reaction to heat stress is to increase cardiac output, sweat production and skin perfusion [8], but there is evidence that in older adults extreme heat leads to a reduction in skin blood flow, which in turn reduces the ability to dissipate heat and negatively affects maintenance of body core temperature [13]. This is relevant with regard to heat tolerance during physical performance when additional heat is produced by the muscles.

Aerobic fitness positively influences heat-tolerance in middle-aged and older adults [14,15]. Although there is evidence that aerobic capacity is reduced in the heat in young people [16], so far no studies on older adults are published.

The aim of this study was to evaluate the feasibility of a test protocol to investigate BP response and aerobic capacity of older adults during exposure to a warm indoor environment. It was hypothesized that BP and aerobic capacity are lower in a hot environment compared with a normal temperature condition and that these changes in BP response and aerobic capacity are more distinct in older adults with low aerobic capacity.

## 2. Methods

### 2.1. Design

BP response and aerobic capacity were assessed in this cross-sectional, experimental study after brief exposure of 60 min to a hot (30 °C) or normal (20 °C) temperature in a climate chamber. The rationale for choosing 20 °C and 30 °C was a population-based inquiry of ambient temperature perceived as comfortable and hot [17] and this includes one condition in (20 °C) and above the thermo-neutral zone (30 °C), each. Both test conditions were assessed in random order with an interval of one week.

### 2.2. Subjects

Twenty-six community-dwelling women, aged 70 years or older, were recruited from a participant list of a previous study on brief exposure to cold temperature [18] after screening for exclusion criteria: (1) not able to follow instructions, (2) presence of uncontrolled cardiac illness, (3) diseases, which significantly influence walking performance, and (4) terminal illness. Only women were included in this pilot-study to counteract possible gender effects. All participants gave written informed consent. The study was approved by the ethical committee of the University of Tuebingen.

### 2.3. Primary Outcomes

Primary outcomes were systolic and diastolic BP and heart rate at rest and during an orthostatic hypotension test. Furthermore, aerobic capacity was a primary outcome.

Systolic and diastolic BP and heart rate were measured in a sitting position by a wrist worn BP monitor (bosco-medistar S, Bosch + Sohn, Jungingen, Germany) automatically inflating the cuff. In addition, an orthostatic hypotension test was performed with 5 min lying supine, then standing up rapidly, and then standing still for 5 min [19]. BP and heart rate were measured every minute during the orthostatic hypotension test. Mean heart rate and BP in supine position as well as minimum BP and maximum heart rate in standing position were calculated as outcome variables of the orthostatic hypotension test.

Aerobic capacity was assessed with the 6-Minute-Walk test [20]. The participants were instructed to walk as far as possible (*i.e*., brisk walking) in a 6 min period, around a 24 m course. A staff member always walked slightly behind the person, without pacing, and announcing the elapsed time every minute. Distance walked in 6 min was recorded.

### 2.4. Secondary Outcomes

Body core temperature and skin temperature were secondary outcomes. Tympanic temperature was measured (Thermoscan 4000, Braun, Kronberg, Germany) as a correlate of body core temperature. Skin temperature was measured (GTH 175/Pt, Greisinger, Regenstauf, Germany) at the proximal backside of the right calf.

### 2.5. Descriptive Variables

Habitual gait speed, self-reported morbidity, fall history and cognition were used as descriptive variables. Habitual gait speed was assessed on a 9 m long instrumented walkway (GAITRite^®^, CIR Systems, Haverton, USA) at 20 °C room temperature. Self-reported morbidity [21] and falls during the previous year (“Did you have a fall during the last 12 months?”) were assessed in an interview. Cognition was screened by the Short Orientation Memory Concentration test [22] with weighted scores >10 suggesting cognitive deficits.

### 2.6. Procedure

Before the measurements in the hot (30 °C) or normal (20 °C) temperature participants were exposed for 30 min to the respective temperature, just sitting on a chair without any physical assessment. Clothing was standardized (trousers, long-sleeved shirt). The room was heated some hours before testing and deviation of ±2 °C was adjusted automatically. Adjustment of humidity was not possible, but humidity was measured and was always between 45% and 50%. During the first 30 min self-reported morbidity, falls history (both at 30 °C condition) and cognition (at 20 °C condition) were assessed. After 30 min of exposure, gait analysis (duration ca. 10 min, only at the 20 °C condition) and the orthostatic hypotension test (*duration ca*. 20 min) were performed. Thereafter, BP and heart rate, which were used to compare the 20 °C and 30 °C condition, were assessed 3 min after the end of the orthostatic hypotension test in a sitting position, because then the women were exposed for about 60 min to the condition and were not physically exerted. Lastly, aerobic capacity was assessed by the 6-Minute-Walk test.

### 2.7. Statistics

Due to the small sample size non-parametric tests were used to describe differences between conditions (Wilcoxon rank-sum test) and associations between parameters (Spearman’s *r*). The significance level of all statistical procedures was set to α = 5% (two-sided). All analyses were conducted using SPSS version 16 software (SPSS Inc., Chicago, IL, USA).

## 3. Results

All women (*n* = 26, median age 75.5 years) completed the measurements without any side-effects. The participants, of whom 35% reported that they had experienced a fall in the previous 12 months, are described in detail in Table 1.

**Table 1:** Baseline characteristics of all participants (*n* = 26 women).

Characteristics	Median	IQR	Minimum–Maximum
Age (years)	75.5	73.0–78.5	71–83
Height (cm)	163	157–168	152–174
Weight (kg)	70	59.8–76.6	46–89
BMI (kg/m^2^)	26.4	22.9–29.0	19.4–33.9
SOMC (0–28)	0	0–4	0–10
Co-morbidities (0–18)	2	1–2	0–7
Habitual gait speed (m/s)	1.23	1.08–1.37	0.81–1.52

IQR = inter-quartile range; BMI = Body Mass Index; SOMC = Short Orientation Memory Concentration test; better score values are underlined.

*BP at rest:* After 60 min of exposure to 30 °C room temperature BP at rest was statistically significant lower when compared with 20 °C room temperature, whereas core and calf skin temperature and heart rate were higher (Table 2).

*Orthostatic hypotension test:* During the orthostatic hypotension test systolic BP decreased in both test conditions (both *p* < 0.001) with 9 (35%) and 11 (42%) participants’ BP dropping more than 20 mmHg in the 30 °C and 20 °C condition, respectively. In contrast, no change in diastolic BP was observed and no difference between the two test conditions was found for the changes in systolic and diastolic BP during the orthostatic hypotension test (Table 2). Heart rate increased after standing up from a lying position (both conditions *p* < 0.001) with a higher increase at the 30 °C condition (Table 2).

*Aerobic capacity:* Compared with the 20 °C condition, the distance walked in 6 min was reduced by 4.9% (29.3 m) after exposure to 30 °C ambient temperature (Table 2). Differences in systolic and diastolic BP response between test conditions were not associated with the distance walked in 6 min (*r* = −0.106, *p* = 0.605 and *r* = −0.059, *p* = 0.775, respectively).

**Table 2:** Differences between 20 °C and 30 °C room temperature of all 26 older women in physiological parameters and aerobic capacity.

Variables	20 °C room Temperature		30 °C room Temperature		*p*
	Median	IQR	Median	IQR	
Sys. BP at rest (mmHg)	143.5	135.3–158.5	133.5	121.5–140.0	<0.001
Dias. BP at rest (mmHg)	88.0	80.0–93.5	80.0	72.3–91.0	<0.001
Heart rate at rest (bpm)	65	61–68	68	64–77	0.016
Core temperature at rest (°C)	36.5	36.0–36.7	36.8	36.6–36.9	<0.001
Calf skin temperature at rest (°C)	31.9	30.7–32.5	34.2	33.7–34.4	<0.001
Sys. BP change (mmHg)	−14	−27–−5	−17	−23–−9	Ns
Dias. BP change (mmHg)	−4	−10–−5	−2	−8–9	Ns
Heart rate change (bpm)	13	6–17	18	10–21	<0.001
6-Minute-Walk (m)	602.1	540.2–635.8	572.8	533.8–641.8	0.008

IQR = inter-quartile range; Sys. = systolic; Dias. = diastolic; BP = blood pressure; change = change from lying to standing position; ns = not significant; negative values indicate a decrease.

## 4. Discussion

The low number of co-morbidities and more than 50% with a perfect cognitive performance test show that the participating older women were still relatively healthy. In addition, a median habitual gait speed of 1.23 m/s indicates a good physical performance [23]. Completion of all measurements and non-occurrence of any negative side effects suggest applicability of the protocol for a planned confirmatory study.

Lower BP and a higher heart rate at 30 °C are physiological response in a hot environment. The results are in line with the results of a study in younger people [10] and confirm our hypothesis that BP is lower in a hot environment also in older people. Since systolic BP dropped in the 20 °C and 30 °C condition after standing up from a lying position, possible problems with orthostatic hypotension related to a lower baseline BP at 30 °C may indicate a higher risk of falling for older adults during heat exposure [11]. The drop of BP during the orthostatic hypotension test was not statistically different between the two test conditions, the increase in heart rate, however, was larger at 30 °C than at 20 °C. It could be hypothesized that BP drop may be also larger at the 30 °C condition in a study with more power due to a larger sample size. The combined fall of BP and rise of heart rate in our study are physiological responses to heat stress and orthostatic stress in older but still relatively healthy participants after a short heat exposure. However in other studies, excess mortality was mainly observed in older adults with poor physical performance during past heat waves [3,4,5,6,7]. In addition, the ability to recover appears to be impaired predominantly in those older adults with regard to orthostatic heart rate response and, to a lesser extent, to systolic BP [24]. Therefore, the capacity to adapt to the heat stress in our test condition may have been worse if typical geriatric patients with multi-morbidity and poly-pharmacy had participated.

Aerobic capacity was lower in the 30 °C condition, which is above the thermo-neutral zone, compared to the 20 °C condition, which is in the thermo-neutral zone. Our results confirm our hypothesis and corroborating the results of a study with younger healthy men [16]. As a consequence, any aerobic exercises, recommended to improve general health condition [25], should be adapted to the environmental temperature. Although no association between endurance capacity and BP response after standing up was found in our study, this may be explained by the small sample size. In general, the results of this pilot-study should be taken with caution and have to be confirmed in a study including a larger sample of older men and women. Furthermore, our protocol of the orthostatic hypotension test (5 min lying, 5 min standing) may be regarded as a methodological limitation, since the use of a tilt table is recommended [26]. We used our protocol because we thought that using your own muscles to stand up shows more ecological validity than a tilt table without muscles involved, provoking a higher drop in BP.

## 5. Conclusions

In conclusion, an adequate physiological response of decrease in BP and increase in heart rate was observed in healthy older women after a brief exposure to a hot environment. However, in older adults with poor physical performance and impaired cardio-vascular response mechanisms these physiological responses are questionable and should be investigated. Furthermore, aerobic capacity of older women was reduced after exposure to hot environmental temperatures, which should be considered when recommending exercise to older people during the summer months.
